# Supplementary material for: Continuous-Wave Pumped Monolayer WS2 Lasing for Photonic Barcoding
Source: Nanomaterials (Basel). 2024 Mar 30;14(7):614. doi: 10.3390/nano14070614 (PMC11013185; doi:10.3390/nano14070614)
Supplement: Supplementary file 1 [file nanomaterials-14-00614-s001.zip › nanomaterials-2921498-supplementary.pdf]

nanomaterials

Article

Continuous-Wave Pumped Monolayer WS_2 Lasing for Photonic Barcoding

Haodong Cheng [1,2,3], Junyu Qu [1,2], Wangqi Mao [3,*], Shula Chen [1,2,*] and Hongxing Dong [3,4,*]

1 Key Laboratory for Micro-Nano Physics and Technology of Hunan Province, College of Materials Science and Engineering, Hunan University, Changsha, Hunan 410082, P.R. China; shuch@hnu.edu.cn (S.C.); haodongc@hnu.edu.cn (H.C.); qujunyu@hnu.edu.cn (J.Q.)
2 Hunan Institute of Optoelectronic Integration, Hunan University, Changsha, 410082, China
3 Key Laboratory of Materials for High-Power Laser, Shanghai Institute of Optics and Fine Mechanics, Chinese Academy of Sciences, Shanghai 201800, China; hongxingd@siom.ac.cn (H.D.); mwangqi@mail.ustc.edu.cn (W.M.)
4 Hangzhou Institute for Advanced Study, Chinese Academy of Sciences, Hangzhou 310024, China
* Correspondence: hongxingd@siom.ac.cn; shuch@hnu.edu.cn; mwangqi@mail.ustc.edu.cn

Supplementary Information for

Continuous-Wave Pumped Monolayer WS_2 Lasing for Photonic Barcoding

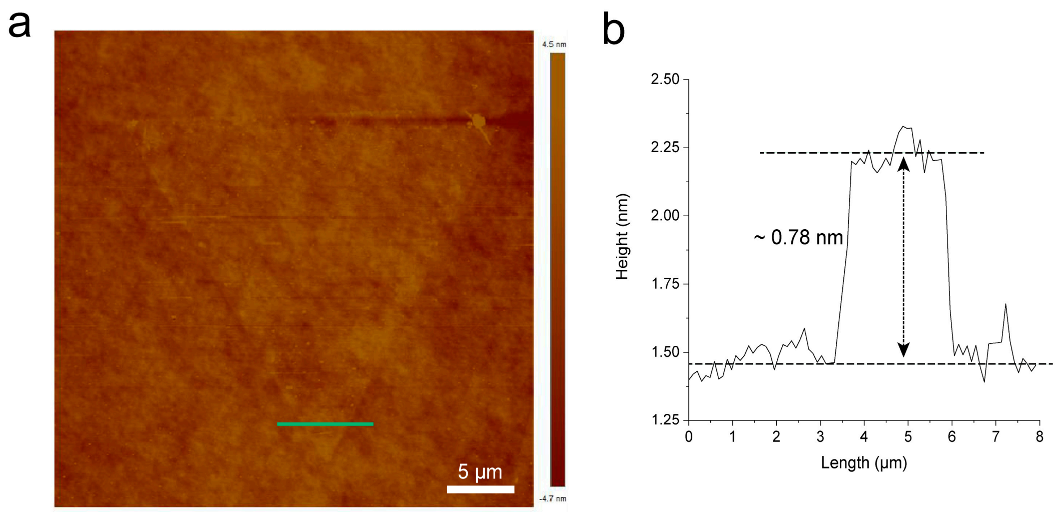

Figure S1. (**a**) AFM image of a monolayer CVD-grown WS_2. (**b**) The height profile curve along the solid green line in (**a**). The thickness of as-grown monolayer WS_2 is about 0.78 nm.

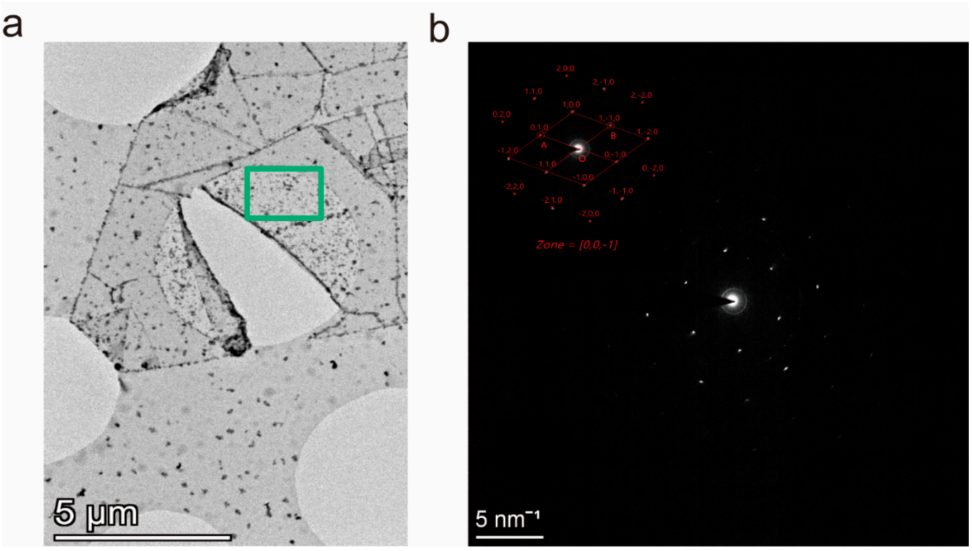

Figure S2. (**a**) A low-magnification TEM image of the triangular WS_2 monolayer transferred onto Cu grids. (**b**) SAED pattern of the WS_2 at the location of the green box in (**a**). Inset: calibration of the SAED pattern.

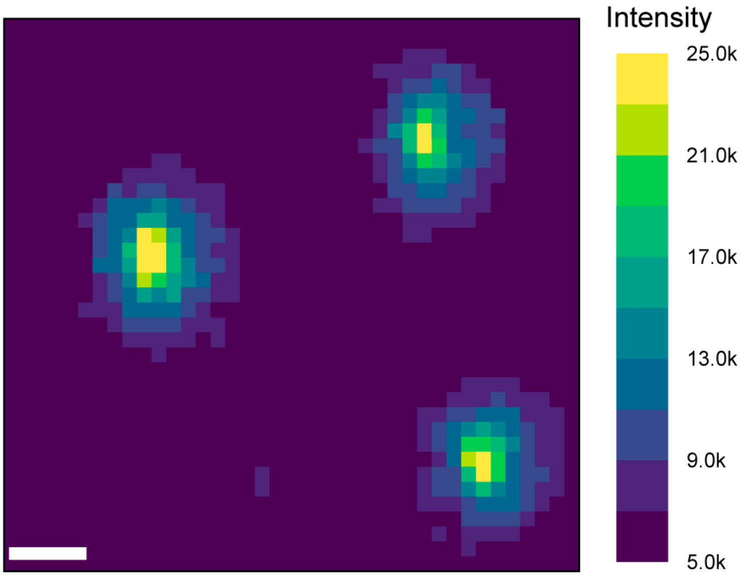

Figure S3. PL mapping image of CVD-grown WS_2 with the external cavities by plotting intensity at 620 nm when the pumping power was 500 W cm^{-2}. Scale bar is 10 μm.

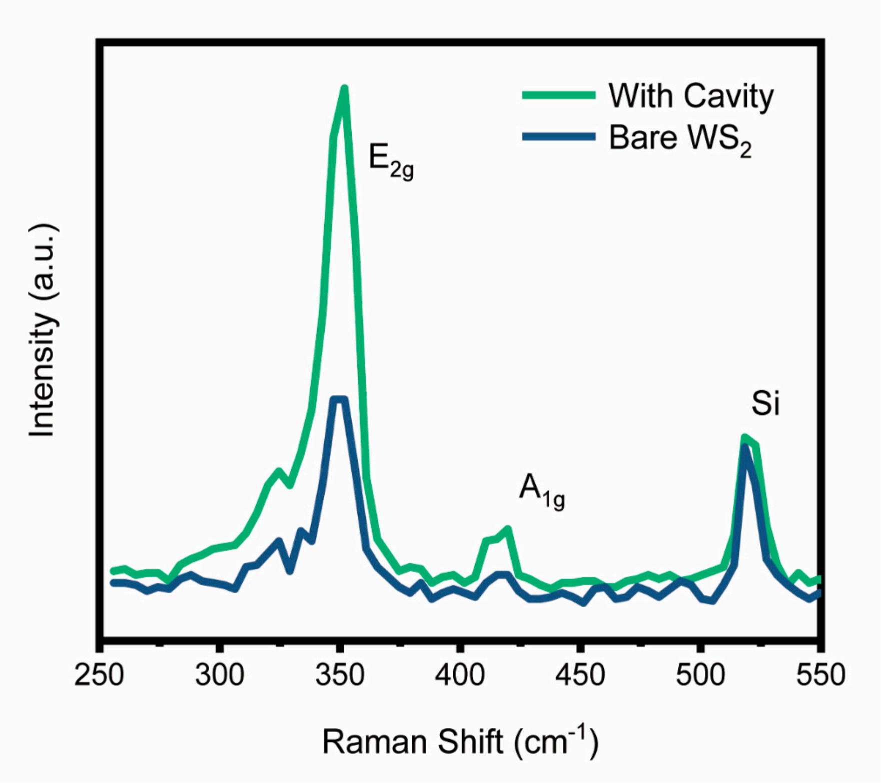

Figure S4. Raman spectra of monolayer WS_2 and WS_2 coupled with the resonant cavity. The difference in wavenumber between E_{2g} and A_{1g} characteristic modes is 62.5 cm^{-1}, which confirms that the CVD-grown WS_2 films are monolayer. The vigorous intensity of Si at 520 cm^{-1} is another evidence of synthesized ultra-thin WS_2.

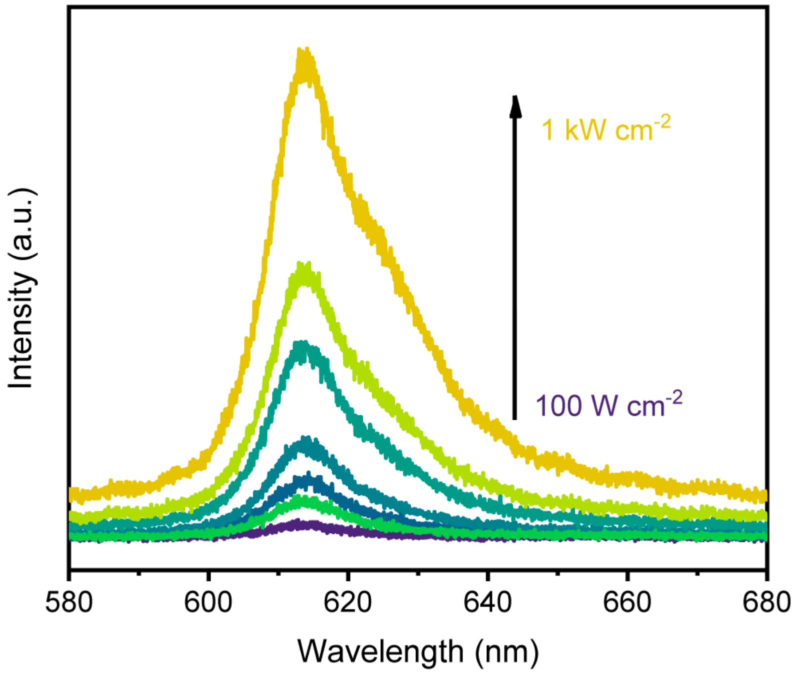

Figure S5. PL spectra of bare WS_2 at different excitation powers. The excitation power is 100 W cm^{-2}, 200 W cm^{-2}, 300 W cm^{-2}, 400 W cm^{-2}, 600 W cm^{-2}, 700 W cm^{-2}, and 1 kW cm^{-2} in order from low to high.
